# Lesion area progression in eyes with neovascular age-related macular degeneration treated using a proactive or a reactive regimen

**DOI:** 10.1038/s41433-023-02652-3

**Published:** 2023-07-01

**Authors:** Mariano Cozzi, Davide Monteduro, Raffaele Antonio Esposito, Kimberly L. Spooner, Samantha Fraser-Bell, Giovanni Staurenghi, Francesco Romano, Matteo Airaldi, Andrew A. Chang, Alessandro Invernizzi

**Affiliations:** 1 Eye Clinic, Department of Biomedical and Clinical Science, University of Milan, Milan, Italy; 2 Sydney Retina Clinic, Sydney, Australia; 3 Faculty of Health and Medicine, Save Sight Institute, University of Sydney, Sydney, NSW Australia

**Keywords:** Outcomes research, Macular degeneration

## Abstract

**Background:**

To compare the change in lesion area over 4 years of follow-up in eyes with neovascular age-related macular degeneration (nAMD) treated with anti-vascular endothelial growth factor (VEGF) agents using either a proactive or a reactive regimen in routine clinical practice.

**Methods:**

This was a multicentre, retrospective comparative study. Totally, 202 treatment-naïve nAMD eyes (183 patients) received anti-VEGF therapy according to a proactive (*n* = 105) or reactive (*n* = 97) regimen. Eyes were included if they had received anti-VEGF injections for a period of at least 4 years and had baseline fluorescein angiography and annual optical coherence tomography (OCT) imaging. Two masked graders independently delineated the lesion’s margins from serial OCT images and growth rates were calculated.

**Results:**

At baseline, the mean [SD] lesion area was 7.24 [5.6] mm^2^ in the proactive group and 6.33 [4.8] mm^2^ in the reactive group respectively (*p* = 0.22). After four years of treatment, the mean [SD] lesion area in the proactive group was 5.16 [4.5] mm^2^ showing a significant reduction compared to the baseline (*p* < 0.001). By contrast, the mean [SD] lesion area kept expanding in the reactive group during the follow-up and was 9.24 [6.0] mm^2^ at four years (*p* < 0.001). The lesion area at 4 years was significantly influenced by treatment regimen, baseline lesion area, and proportion of visits with active lesions.

**Conclusions:**

Eyes treated using a reactive strategy had an increased lesion area and worse visual outcomes at 4 years. By contrast, the proactive regimen was associated with fewer recurrences of active disease, shrinkage of the lesion area, and better vision at four years.

## Introduction

Neovascular age-related macular degeneration (nAMD) is an irreversible retinal disease characterized by the presence of exudative macular neovascularization (MNV) [1] that causes a progressive decline of visual function in the elderly population if not treated promptly [2]. In the last two decades, vascular endothelial growth factor (VEGF) inhibitors have been proven as an effective treatment for this condition and have become the first-line therapy option to prevent severe visual deterioration [3].

The randomized controlled trials (RCTs) which led to the approval of anti-VEGF drugs demonstrated the efficacy of fixed dosing regimens with monthly or bi-monthly intravitreal injections [4, 5]. However, to reduce the treatment burden more recent studies have investigated different treatment strategies for the management of nAMD [6, 7].

The two most widely adopted regimens in clinical practice nowadays are the *pro-re-nata* (PRN) and treat-and-extend (T&E). The first is designed to treat only when reactivation of the lesion is observed on structural optical coherence tomography (OCT) and/or new hemorrhage is observed clinically. The second is a proactive, individualized dosing strategy whereby functional and anatomic stability are the key features that act to extend injection intervals. Eyes treated using a reactive or PRN approach receive fewer injections but almost invariably show worse long-term outcomes than those treated with a T&E regimen [8, 9].

There is increasing evidence that less fluid fluctuation is associated with better vision [10, 11]. This could be a possible explanation for the better outcomes seen in eyes treated with T&E as the proactive strategy allows for fewer exudation recurrences [12]. However, other factors are known to influence visual outcomes regardless of the treatment regimen like the size of the lesion, the fibrosis [13], and the development of macular atrophy (MA). In a previous study, our group demonstrated no effect of the treatment regimen on the incidence or progression of MA [14]. However, there are limited data on the effect of different treatment strategies on the lesion area.

The aim of this study was to assess the change in lesion area in treatment-naïve patients affected by nAMD treated with anti-VEGF agents using a proactive regimen compared to a reactive regimen over 4 years and to correlate them with clinical outcomes.

## Methods

### Protocol

This was a retrospective, multicentre study sharing part of the database with the previously published MANEX study [14]. Consecutive patients undergoing anti-VEGF therapy for nAMD from two retinal clinics in Sydney, Australia, and Milan, Italy, were included. These clinics were chosen because nAMD patients were managed differently in the two sites as per standard clinical practice and internal guidelines. In particular, patients from the clinic based in Sydney were treated with a proactive regimen while those from Milan received reactive treatment. Detailed inclusion criteria have been reported previously [14]. In brief, treatment-naïve eyes with nAMD and 4 years of follow-up were included in the analysis. Additionally, eyes with low-quality fundus fluorescein angiography (FFA) at baseline, ungradable OCT scans, or with a lesion area exceeding the OCT volume at any visit were excluded from the analysis. To be included in the study, the scan protocol consisted of at least 20 × 15° with no less than 19 B-scans per volume. Moreover, all the consecutive scans were performed with the eye tracking activated during the baseline visit. The study was conducted in accordance with the Declaration of Helsinki. The protocol was approved by the Independent Ethics Committee/Institutional Review Board at each site. The Institutional Review Board approved a waiver of consent due to the study’s retrospective nature.

### Data acquisition

Medical records were reviewed in order to obtain demographic data, visual acuity (VA) reported in early treatment diabetic retinopathy study (ETDRS) letter score, and the number of intravitreal anti-VEGF injections administrated with the corresponding drug. Additionally, we collected disease control data (DCD), defined as the percentage of visits with active MNV.

In both sites, all treatment-naïve patients received 3 monthly intravitreal injections of anti-VEGF agents followed by either a proactive or a reactive protocol. Patients followed by the Sydney Retina Clinic, Sydney, Australia, were treated with T&E. Patients followed by the Eye Clinic, Department of Biomedical and Clinical Science, Luigi Sacco Hospital, University of Milan, Milan, Italy, were treated with a reactive approach. The two treatment strategies were applied as follows:

- Proactive strategy: 3 monthly loading doses of anti-VEGF injections. If no activity signs were detected by OCT or a new hemorrhage was seen at funduscopic examination, the interval of the following injection was extended by 2 weeks, up to a maximum of 12 weeks. If activity or hemorrhage was present, the interval was decreased by two weeks to a minimum of 4 weeks.
- Reactive strategy: 3 monthly loading doses of anti-VEGF injections followed by subsequent injection administrated only if a drop in VA, new hemorrhage, or activity detected by OCT occurred. In the absence of re-treatment criteria, patients were scheduled for follow-up in 4–8 weeks. The PRN clinic was run as a two-stop service (monitoring and injection on different days, typically scheduled within one week).

### Images analysis

Spectral Domain OCT scans obtained with Heidelberg Spectralis (Heidelberg Engineering, Heidelberg, Germany) were used for the analysis. In particular, the volume collected at the baseline and each follow-up volume performed yearly were carefully reviewed for the purpose of this study. In case of poor quality of the scans or absence of OCT image, the first available visit within three months from the yearly examination was used. Overall, we included five different OCT volumes per eye (from baseline to year 4 visit).

Baseline multimodal clinical images were graded by two independent readers (K.S. and M.C.) to classify MNV lesion type based on the most recent nAMD consensus nomenclature (Type 1, Type 2, Type3 or Polypoidal Choroidal Vasculopathy) [1]. Discordant cases were reviewed and assessed by a senior clinician (A.C. and G.S.). Central subfield retinal thickens are automatically calculated by the inbuilt OCT reviewing software (Heidelberg Eye Explorer, software version 1.9.10.1, Heidelberg Engineering, Heidelberg, Germany) as the distance between the internal limiting membrane to the Bruch’s membrane within the central 1 mm of the ETDRS grid subfield was collected. Segmentation artifacts were manually corrected when necessary.

The lesion area was calculated by analyzing the combined OCT B-scans and the topographic correspondence on the near-infrared reflectance (NIR) image. This approach was necessary as FFA images, which have been traditionally used for this purpose in RCTs [15, 16] were available only at baseline. To consistently comprise the presence of the entire lesion (either below or above the retinal pigment epithelium), we included any pigment epithelial detachment (PED) with signs of fibrovascular tissue underneath. In addition, subretinal hyperreflective material (SHRM) was counted as it is characterized by fibrovascular tissue and potentially other components such as blood, fluid, and lipids in the subretinal space [17]. Hyperreflective material between focal RPE defects and inner retinal layers was also considered to include early stages of type 3 MNV [18].

**Fig. 1:**
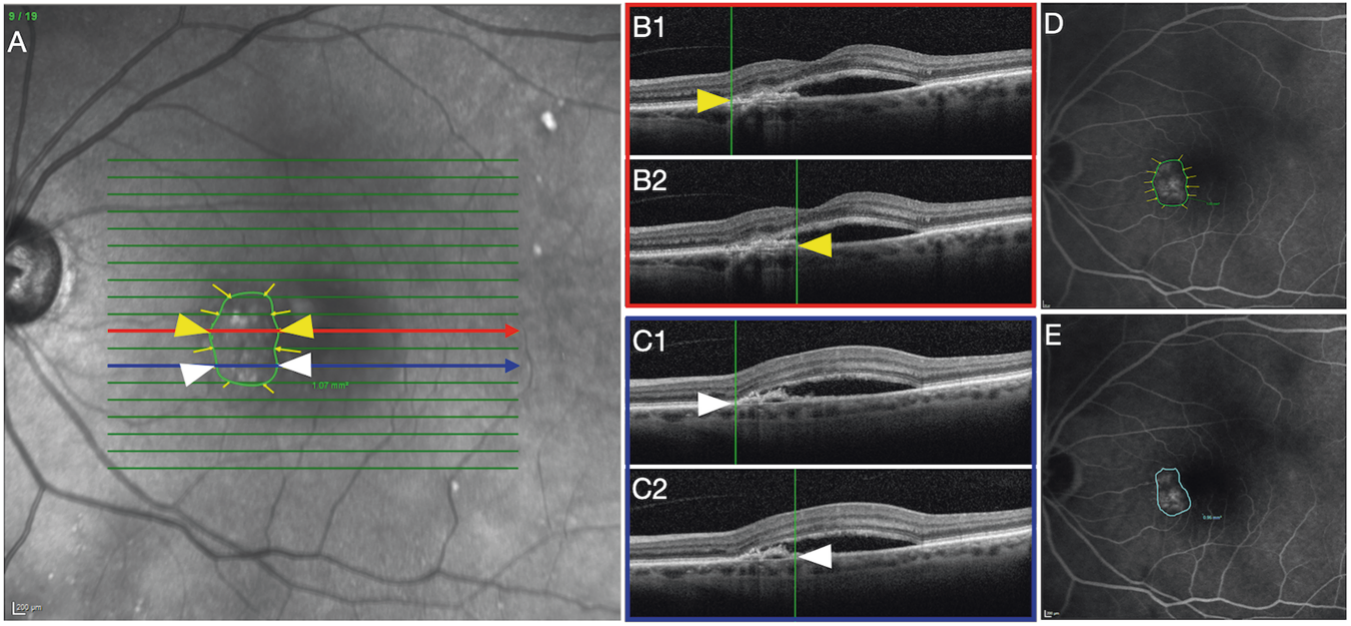
OCT-based strategy used to calculate lesion area. **A** Near-infrared reflectance (NIR) showing the volume scan pattern superimposed with the presence of yellow arrows applied by the grader based on the correspondence B-Scans. **B**, **C** Two sample optical coherence tomography (OCT) B-scans are presented to demonstrate how the reader defined MNV lesion area borders based on the B-scan. B1 and C1 show the left edges of the lesion area while B2 and C2 exhibit the right limit of the lesion. Arrowheads reveal how the analogs points correspond between NIR and OCT. **D** Fundus fluorescein angiography (FFA) shows the overlayed lesion area obtained connecting the marks on NIR. **E** Macular neovascularization lesion area measured on the early phase FFA.

In brief, each B-scan of a macular volume was singularly assessed to determine the presence of the lesion and its margins. If elements considered part of the lesion were identified the grader marked their edges along the b-scan on the combined 30 × 30° NIR image. This was possible as the inbuilt OCT reviewing software allows for topographically localized OCT positions on the NIR image. The OCT features considered part of the lesion and used to delineate the margins were: fibrovascular PED, mixed PED, and SHRM. Intraretinal fluid, subretinal fluid, macular atrophy, drusen, drusenoid PED, and serous PED were excluded from the lesion area count. The MNV edges identified on the OCT scans and reported on the NIR image were then connected together using the “draw region” tool of the inbuild reviewing software to obtain the lesion area (Fig. 1).

In order to prove the reliability of this novel approach, we also calculated the area of the lesions at baseline directly drawing the margins using the “draw region” tool on the FFA images [15, 16] and we compared the size of the lesion obtained with the two methods. In addition, all OCT-based measurements were performed at all visits by two independent readers (K.S. and D.M.), and in case a difference of more than 20% was found between measurements, arbitration through open adjudication was performed. In cases where an agreement was not achieved, a third-grader established a resolution (AI). An average of the two measurements from the two readers was used for comparison with FFA-based lesion size calculation and to perform the subsequent statistical analysis.

### Statistical analysis

The normal distribution of the data was tested for all quantitative variables before the analysis. Results are reported as mean and standard deviation (SD) for continuous variables, while categorical variables are presented as numbers and percentages where appropriate. Baseline demographic and clinical characteristics were compared between the two treatment strategies using *t*-tests, Wilcoxon ranks sum tests, and Chi-square tests where applicable. The reliability of the OCT-based assessment of the lesion area was tested by comparing the measurements obtained with this method with the gold standard based on FFA by means of the intraclass correlation coefficient (ICC). Additionally, the inter-rater agreement was assessed between the two independent graders.

Given the repeated measures structure of the data, a two-way mixed ANOVA was used to compare the main effects of time and treatment protocol on VA, lesion Area, number of OCT per year, number of anti-VEGF treatments per year, and proportion of visits graded as active per year. Post hoc pairwise *t*-tests were then used for comparisons between the two groups at each visit.

The lesion area growth over four years of treatment was compared between proactive and reactive regimens using longitudinal generalized linear mixed-effects models, with the interaction between treatment regimen and time as the primary predictor variable.

The longitudinal model was also adjusted for age, baseline lesion area, type of MNV lesion, the total number of injections, anti-VEGF agent and DCD during the follow-up, and nesting of outcomes within patients with bilateral disease as random effects.

*P*-values from pairwise comparisons were adjusted for using the Holm–Bonferroni correction. A *p*-value < 0.05 was considered statistically significant. Analyses were conducted in R software version 4.1.2 (R Project—The R Foundation for Statistical Computing, Vienna, Austria).

## Results

Of 264 treatment-naïve eyes included in the MANEX study, 202 met the inclusion criteria and were included in the study. Among the excluded eyes, 21 had ungradable scans, 23 lesions exceeded the scan volume area and 18 had low-quality FFA. One-hundred-seventy-two eyes were first treated with ranibizumab, 26 with aflibercept, and the remaining 4 eyes with bevacizumab. A total of 105 eyes were treated according to the proactive regimen, while 97 eyes were treated with a reactive approach.

### Baseline characteristics

The overall mean [SD] age was 78.6 [8.8] years (range 55–95) and 74.3 [8.1] years (range 52–91) for the proactive and reactive groups, respectively, with a slightly significant difference between the two cohorts. Males comprised 53% of the proactive group and 36% of the reactive group. The groups were well balanced at baseline for VA (*p* = 0.58). The distribution of MNV lesion type also was similar between the two groups. The baseline characteristics of the two treatment cohorts are summarized in Table 1.

**Table 1:** Baseline demographic and ocular characteristics of the study patients.

Baseline demographic and ocular characteristics			
	Proactive (*n* = 105)	Reactive (*n* = 97)	*p* Value*
Age (yrs), mean ± SD	78.6 ± 8.8	74.3 ± 8.1	*p* < 0.001
*Gender, n (%)*			
Male	56 (53%)	35 (36%)	*p* = 0.01
Female	49 (46%)	62 (64%)	
VA (ETDRS score), mean ± SD	66.6 ± 14.1	65.6 ± 14.7	*p* = 0.58
CMT (µm), mean ± SD	414.5 ± 128.1	419.6 ± 137.9	*p* = 0.79
*MNV lesion type, n (%)*			
Type 1	59 (56%)	60 (62%)	*p* = 0.61
Type 2	21 (20%)	15 (16%)	
Type 3 (RAP)	14 (13%)	13 (13%)	
PCV	11 (11%)	9 (9%)	
Lesion area (mm^2^), mean ± SD	7.24 ± 5.6	6.33 ± 4.8	*p* = 0.22

SD standard deviation, VA visual acuity, *ETDRS* early treatment diabetic retinopathy study, *CMT* central macular thickness, *MNV* macular neovascularization, *RAP* retinal angiomatous proliferation, *PCV* polypoidal choroidal vasculopathy, * *t*-test/Wilcoxon ranks sum test/chi-square test.

### Lesion area

The agreement between the OCT-based assessment of the lesion area and the standard FFA was found to be “good” to “excellent”, ICC = 0.85; 95% CI [0.81–0.92]. The agreement between the two independent readers of the lesion area OCT-based measurement at baseline was found to be “excellent”, ICC = 0.95; 95% CI [0.90–0.97].

At baseline, the mean [SD] lesion area was 7.24 [5.6] mm^2^ in the proactive group and 6.33 [4.8] mm^2^ in the reactive group (*p* = 0.22).

After the first year of treatment, the two groups showed opposite behaviors. The proactive group showed a significant decrease in the lesion area (mean [SD] change from baseline −1.19 [4.13] mm^2^ *, p* = 0.005) while the reactive cohort demonstrated significant enlargement of the lesions (mean [SD] change from baseline 0.73 [3.42] mm^2^*, p* = 0.039).

**Fig. 2:**
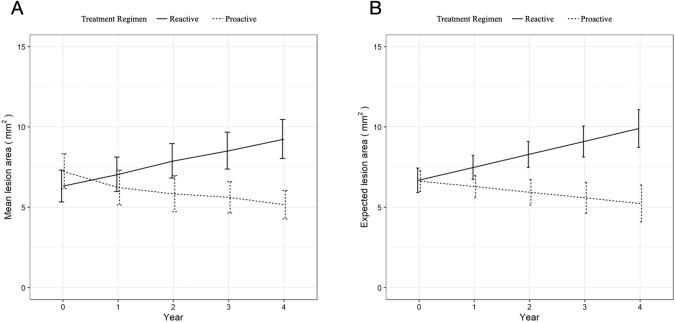
Lesion area progression in the two study groups. **A** Overall trend of the lesion area in the two study groups during the entire follow-up period. **B** Longitudinal predictive model for the lesion area changes in the two cohorts.

After 4 years of treatment, the mean [SD] lesion area in the proactive group was 5.16 [4.5] mm^2^, remaining significantly smaller compared to baseline (*p* < 0.001). By contrast, the mean [SD] lesion area kept growing in the reactive group during the follow-up and was significantly larger at four years (9.24 [6.0] mm^2^, *p* < 0.001) compared to baseline. Lesion area values at yearly time points and comparisons between the two groups are reported in Table 2. The overall trend of the lesion area in the two groups during the entire follow-up is represented in Fig. 2A.

**Table 2:** Best correct visual acuity and lesion area at yearly time points in the two treatment groups.

	BCVA				Lesion Area			
	ETDRS letters, mean (SD)				mm^2^, mean (SD)			
	Proactive	Reactive	twmANOVA, *p* Value^#^	*p* Value*	Proactive	Reactive	twmANOVA, *p* Value^#^	*p* Value*
Baseline	66.6 (±14.1)	65.6 (±14.7)	0.013	0.576	7.2 (±5.6)	6.3 (±4.8)	<0.001	0.219
Year 1	69.2 (±14.3)	69.1 (±13.6)		0.869	6.3 (±5.5)	7.1 (±5.3)		0.287
Year 2	69 (±14.6)	67.4 (±15.1)		0.531	5.8 (±5.8)	7.9 (±5.3)		0.01
Year 3	68.1 (±14.9)	65.7 (±14.7)		0.304	5.7 (±4.9)	8.5 (±5.6)		<0.001
Year 4	68.3 (±13.6)	62.1 (±16.5)		0.005	5.2 (±4.5)	9.3 (±6)		<0.001

*BCVA* best correct visual acuity, *SD* standard deviation, ^#^repeated measure, two-way mixed ANOVA (see Methods); * pairwise *t*-test.

The multivariate analysis identified the final lesion area significantly influenced by the interaction of time and treatment strategy, with a significant decrease of expected lesion area in the proactive treatment regimen over time (beta = −1.172, *p* < 0.001), baseline lesion area, and a total number of injections. None of the other tested actors including age, lesion type, anti-VEGF agent, and total DCD had a significant effect on the lesion area at four years. A graphic representation of the longitudinal predictive model for the lesion area change in the two groups is reported in Fig. 2B and the longitudinal predictive model for the lesion area changes divided by lesion type is available in Supplementary Fig. 1.

Beta coefficients and corresponding *p*-values for the longitudinal mixed-effects model are reported in Supplementary Table 1.

### Visual acuity

Mean [SD] VA significantly increased from baseline to year 1 both in the proactive group (from 66.7 [14.1] to 69.2 [14.3] letter score, *p* = 0.01) and in the reactive group (from 65.6 [14.8] to 69.1 [13.6] letter score, *p* = 0.01). At 4 years the mean [SD] VA was still +1.5 letters compared to the baseline in the proactive group. By contrast, the reactive group’s initial improvement was not maintained, which incurred a significant decline of −3.5 letters at year 4 (*p* = 0.01). VA values at yearly intervals and comparisons between the two groups are reported in Table 2.

### Injections number, OCT visits, and disease activity

Eyes in the proactive cohort received a significantly higher number of anti-VEGF injections during the entire follow-up period compared to those in the reactive group (33.6 [9.5] vs 17.8 [6.1] injections; *p* < 0.001). A similar trend was observed in the number of OCT assessments per eye between the two cohorts. In fact, the eyes treated with the proactive approach were scanned more times than those in the reactive group (36.7 [8.8] vs 26.1 [8.5] OCT scans; *p* < 0.001). The detailed comparisons for each year of follow-up are reported in Supplementary Table 2.

The mean [SD] percentage of visits with a lesion graded as “active” was 18.8 [22.5]% in the proactive group and 54.1 [28.1]% in the reactive group, with the difference between the two being statistically significant (*p* < 0.001). The detailed proportion of visits graded as active between the two groups defined as DCD is summarized in Supplementary Table 2.

## Discussion

**Fig. 3:**
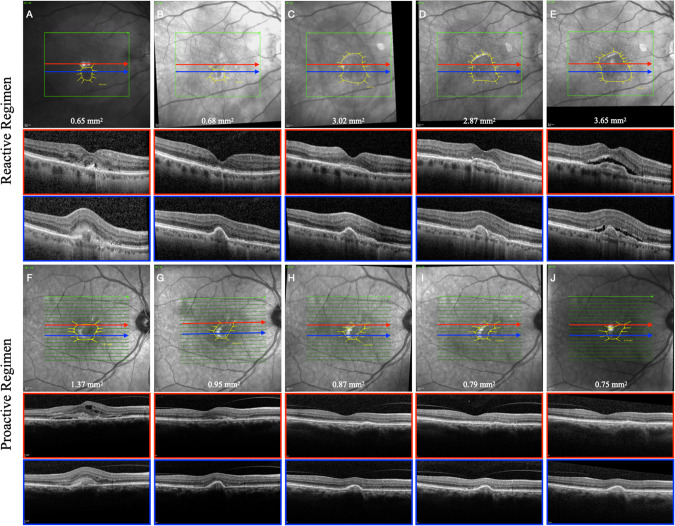
Clinical example of two eyes affected by type 2 MNV included in the study. Example of type 2 macular neovascularization in two patients affected by neovascular age-related macular degeneration under treatment with anti-vascular endothelial growth factor following a reactive and a proactive regimen. **A**, **F** Baseline; **B**, **G** year 1; **C**, **H** year 2; **D**, **I** year 3; **E**, **J** year 4.

Our study demonstrated a difference in the progression of lesion area between the two most widely used treatment regimens in nAMD. In particular, we observed that the MNV lesion area steadily increased during the entire duration of the study in eyes treated with a reactive approach. By contrast, the proactive treatment strategy was associated with a reduction in lesion area at year one compared to baseline and prevented further growth over time. This difference was accompanied by a lower disease activity rate and better visual outcome in the proactive group compared to the reactive one. Two examples of this tendency are presented in Fig. 3.

These findings suggest that a proactive treatment, avoiding activity fluctuation helps constrain enlargement of the lesion area. This supports previous reports that disease relapses are often accompanied by the sprout of new choroidal vessels [19, 20], inevitably resulting in an increase in the lesion area [21].

Interestingly, Miere et al. quantified MNV changes in eyes with neovascular AMD undergoing a monthly loading phase and observed a significant decrease in the lesion area after each monthly injection [22]. Different OCTA studies have also reported lesion area progression in type 1 MNV treated with a PRN regimen at the end of the observation period [23, 24]. These pieces of evidence support the idea that preventing recurrences allows for the reduction and control of the lesion area.

Our findings on the effect of reactive regimen on the lesion area are partially in contrast with the angiographic endpoints presented in the HARBOR study which reported a decrease from baseline in total MNV area after 12 months of treatment in all treatment groups including those receiving PRN injections [16]. The discordance could have several explanations. First, the lesion area was measured in disk areas on fluorescein angiography in RCTs, a much rougher method compared to the pixel-based we used in our study. Secondly, RCTs and retrospective real-world studies often show differences in the outcomes related to the absence of predetermined criteria for retreatment in a real-world context [25]. Lastly and more importantly, longer follow-up usually allows us to appreciate time-dependent effects like lesion growth better. In fact, the 5-year CATT follow-up study unveiled an increase in the mean total lesion area compared with that at 2 years [11, 15], and a larger MNV area was associated with worse VA (*p* < 0.001) [26].

Visual function is almost invariably affected in nAMD. Treatment with anti-VEGF agents limits the damage induced by MNVs to the retina [27, 28]. Despite this, many eyes experience long-term vision loss due to macular atrophy and fibrosis [29, 30]. Retinal thickness fluctuations have also been associated with poorer visual function and an increased risk of fibrosis [31]. In the MANEX study, we demonstrated no effect of the treatment regimen on atrophy development, ruling it out as a possible explanation for the better outcomes achieved by a proactive over a reactive strategy [14]. By contrast, our current findings show a clear relationship between the treatment regimen and the lesion growth helping to explain why fluctuations have been associated with worse outcomes [11]. In fact, not only do relapses stretch the retinal structures impairing function and increasing the risk of hemorrhage, but also allow for the progressive expansion of the lesion [21].

In our analysis, the proactive cohort received nearly twice as many injections as the reactive group during the four years follow-up period. This is consistent with most reports using T&E or PRN regimens in routine clinical practice [32, 33]. In particular, our data are representative of the two involved countries. For instance, the mean treatments in the first two years reported from the Milan site are in line with aggregate data obtained from Italy, Germany, and France [33].

A strict PRN regimen with assessment every 4 weeks remains mostly unachievable in clinical practice for both patients and clinical staff inevitably ending up in a more “relaxed” regimen, unfortunately resulting in delayed re-assessments, more relapses, and worse outcomes. By contrast, a T&E regimen better fits with real-world needs, reducing the number of injections compared to fixed regimens, but still assuring comparable outcomes [6].

This study has several limitations that need to be acknowledged. Some of these are derived from the MANEX study and were extensively discussed in the previous report. In particular, its retrospective nature and the inevitable selection bias of enrolled patients, excluding those who responded exceptionally well or very poorly, must be considered when interpreting our findings. In addition, patients experiencing an excessive growth of the lesion exceeding the area comprised within the OCT volumes had to be excluded, limiting the applicability of our results only to eyes with smaller lesions. The lack of yearly FFA and OCTA imaging forced us to use a novel structural OCT-based method to delineate the lesion margins and calculate the area. We tried to mitigate this limitation by comparing our measurements with those obtained on the FFA at baseline and repeating all measurements twice by two independent experienced retinal specialists.

In conclusion, this study has offered a possible new explanation for the superior outcomes obtained by proactive over reactive regimens. The reactive strategy was associated with a higher proportion of visits with active disease, progressive increase in lesion area, and worse visual outcomes in the long term. By contrast, the proactive regimen characterized by limited lesion recurrences was associated with shrinkage of the lesion and better vision at four years.

## Summary

### What was known before

- The lesion area is associated with visual acuity in neovascular age-related macular degeneration (nAMD).
- Less fluid fluctuation is associated with better vision—better outcomes are seen in eyes treated with proactive strategy as fewer exudation recurrences.

### What this study adds

- These findings suggest that a proactive treatment, avoiding activity fluctuation, helps constrain the enlargement of the lesion area.
- This study offered a possible new explanation for the superior outcomes obtained by proactive over reactive regimens.

### Supplementary information

Supplemental Table 1

Supplemental Table 2

Supplemental Figure 1

## Data Availability

The data that support the findings of this study are available upon request.
