# Supplementary material for: Lesion area progression in eyes with neovascular age-related macular degeneration treated using a proactive or a reactive regimen
Source: Eye (Lond). 2023 Jul 1;38(1):161–7. doi: 10.1038/s41433-023-02652-3 (PMC10764886; doi:10.1038/s41433-023-02652-3)
Supplement: Supplementary file 1 — Supplemental Table 1 [file 41433_2023_2652_MOESM1_ESM.docx]

Supplementary information is available at “Eye Journal’s website” at the end of the article and before the references.

**Supplementary Table 1**: Beta coefficients and corresponding *p*-values for the longitudinal mixed-effects model applied to the lesion area changes.

|  | β Coefficient | Standard Error | *p* value |
| --- | --- | --- | --- |
| (Intercept) | -1.577 | 1.274 | 0.217 |
| Proactive Strategy | 0.085 | 0.550 | 0.878 |
| Year | 0.740 | 0.121 | **<0.001** |
| Year x Strategy (Proactive) | -1.168 | 0.167 | **<0.001** |
| Baseline Age | 0.010 | 0.015 | 0.519 |
| Baseline Area | 0.883 | 0.022 | **<0.001** |
| Type 2 MNV | 0.185 | 0.317 | 0.559 |
| Type 3 MNV | -0.003 | 0.455 | 0.994 |
| PCV | -0.247 | 0.448 | 0.581 |
| Total injections | 0.055 | 0.016 | **<0.001** |
| Aflibercept | -0.592 | 0.356 | 0.097 |
| Bevacizumab | 0.559 | 0.499 | 0.263 |
| Total DCD | -0.397 | 0.731 | 0.587 |
| MNV: Macular Neovascularization; PCV: Polypoidal Choroidal Vasculopathy; DCD: disease control data | | | |
