# Supplementary material for: Lesion area progression in eyes with neovascular age-related macular degeneration treated using a proactive or a reactive regimen
Source: Eye (Lond). 2023 Jul 1;38(1):161–7. doi: 10.1038/s41433-023-02652-3 (PMC10764886; doi:10.1038/s41433-023-02652-3)
Supplement: Supplementary file 2 — Supplemental Table 2 [file 41433_2023_2652_MOESM2_ESM.docx]

Supplementary information is available at “Eye Journal’s website” at the end of the article and before the references.

**Supplementary Table 2**: Injections number in the two treatment groups with the proportion of visits graded as active.

|  | **Injections** | | | |  | **Disease Control Data** | | | |
| --- | --- | --- | --- | --- | --- | --- | --- | --- | --- |
|  | mean (SD) | | | |  |  |  |  |  |
|  | **Proactive** | **Reactive** | **twmANOVA, p Value^#^** | **p Value*** |  | **Proactive** | **Reactive** | **twmANOVA, p Value^#^** | **p Value*** |
| Year 1 | 10.02 (±1.9) | 5.81 (±1.8) | <.001 | <.001 |  | 35.7% (±23%) | 52.9% (±28%) | <.001 | <.001 |
| Year 2 | 8.80 (±3.1) | 4.06 (±2.3) |  | <.001 |  | 18.2% (±23%) | 53.7% (±29%) |  | <.001 |
| Year 3 | 7.71 (±3.2) | 4.18 (±2.1) |  | <.001 |  | 11.1% (±17%) | 57.4% (±26%) |  | <.001 |
| Year 4 | 7.03 (±3.3) | 3.70 (±1.9) |  | <.001 |  | 10.3% (±16%) | 52.4% (±28%) |  | <.001 |
|  | ^#^ repeated measure, two-way mixed ANOVA (see Methods); * pairwise t-test | | | | | | | | |
