# Supplementary material for: Lesion area progression in eyes with neovascular age-related macular degeneration treated using a proactive or a reactive regimen
Source: Eye (Lond). 2023 Jul 1;38(1):161–7. doi: 10.1038/s41433-023-02652-3 (PMC10764886; doi:10.1038/s41433-023-02652-3)
Supplement: Supplementary file 3 — Supplemental Figure 1 [file 41433_2023_2652_MOESM3_ESM.docx]

Supplementary information is available at “Eye Journal’s website” at the end of the article and before the references.


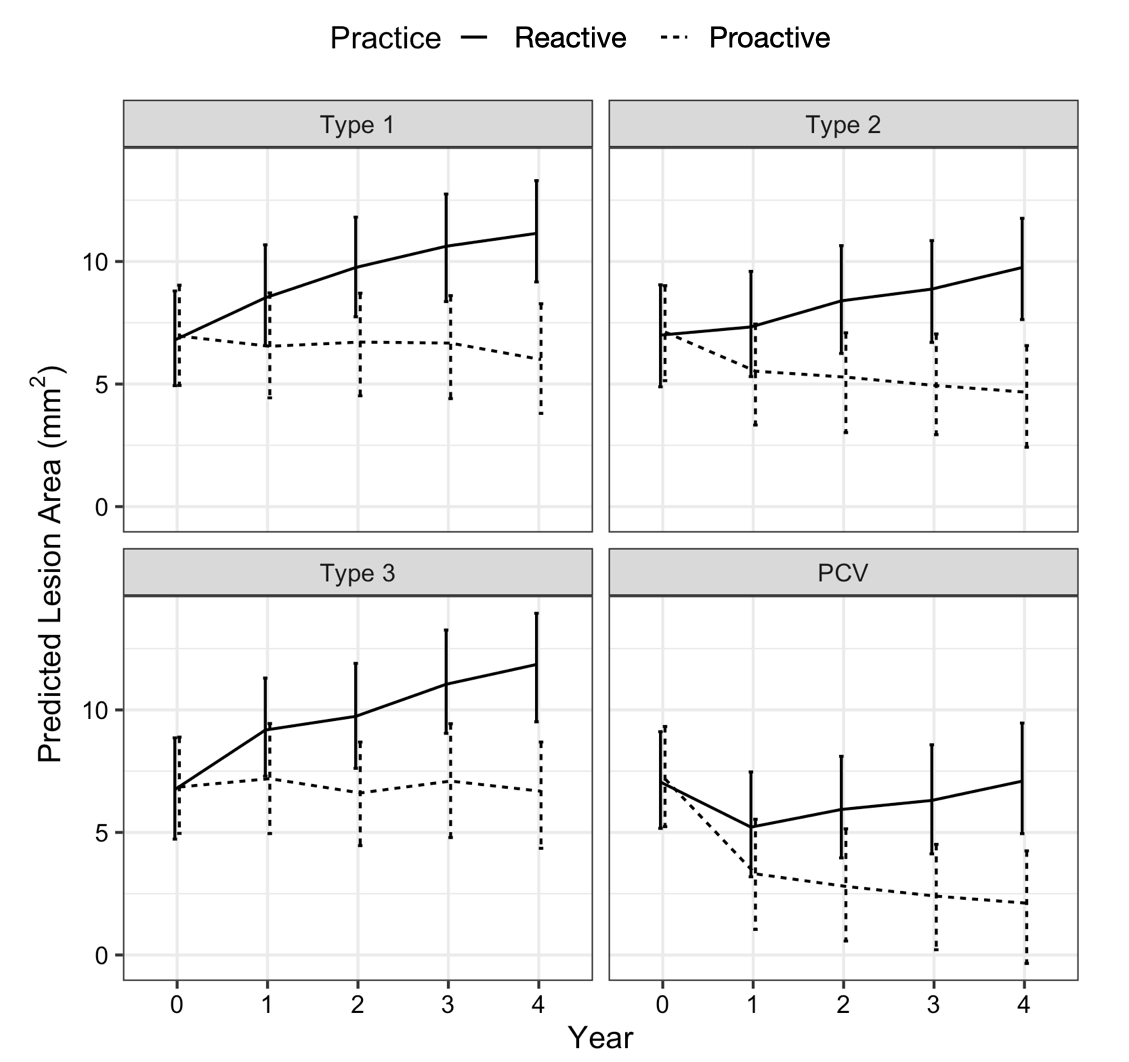


**Supplemental figure 1**: Longitudinal predictive model for the lesion area changes in the two cohorts divided by lesion type.
